# Foix-Chavany-Marie syndrome with an unusual presentation: case report of a stroke with acute trismus

**DOI:** 10.1007/s10072-026-09107-z

**Published:** 2026-05-14

**Authors:** Jonas Ranke, Torben Sachs, Josephin Damm, Julian Klingbeil

**Affiliations:** 1 Department of Neurology, Martin-Luther-University Halle-Wittenberg, Halle (Saale), Germany; 2 Department of Radiology, Martin-Luther-University Halle-Wittenberg, Halle (Saale), Germany

## Introduction

Foix-Chavany-Marie syndrome (FCMS), named after the French authors who originally described the condition as diplegia facio-linguo-masticatorica [1], is a rare form of pseudobulbar palsy, characterized a by a loss of voluntary control over the caudal cranial nerves. It leads to severe impairment of swallowing and speaking while reflectory movements such as laughing are preserved. Most of the nearly 200 cases of FCMS described so far are caused by bilateral opercular strokes. We report an unusual case in which acute trismus initially masked the classical presentation of FCMS.

## Case report

A 64-year-old female patient was admitted to our hospital due to acute onset brachiofacial hemiparesis of the left side and anarthria. Prior to the new deficits, she had lived with a mild residual aphasia and mild paresis of her right arm after a left hemispheric, ischemic stroke several years before. Secondary prevention consisted of acetylsalicylic acid, clopidogrel, rosuvastatin and ezetimibe. On admission, we saw an obese patient with moderate hemiparesis and hemihypesthesia of the left extremities. The most striking finding was severe trismus with markedly increased jaw tone, preventing both active and passive mouth opening. The total NIHSS score was 12. Because of the pronounced trismus, sufficient examination of the oral cavity regarding a minor oral bleeding already present on admission and history taking were initially difficult.

In the cerebral computer tomography with angiography and perfusion imaging, we saw a distal M2 segment occlusion of the right middle cerebral artery with a hypoperfusion in CBV and CBF imaging with preserved mismatch and only incipient demarcation of the central area. Due to the perfusion mismatch and the pronounced clinical symptoms, we initiated an intravenous thrombolysis with 81 mg of rtPA. Treatment was delayed (door-to-needle time 103 min) due to simultaneous emergency imaging of a polytrauma patient and the need for otorhinolaryngological evaluation of the oral bleeding. No significant intraoral, nasal or pharyngeal bleeding source could be objectified and the by that time regredient bleeding was considered minor, most likely caused by mucosal injury due to accidental biting during the acute jaw spasm. A luxation of the temporomandibular joint was excluded in the computer tomography. Due to the distal localization of the thrombus in a non-dominant M2 branch, interventional reperfusion was not performed. Initially, trismus was considered possibly ictal or stress-related and treatment with lorazepam and ketamine was attempted without effect. Regarding the hyperacute treatment setting, unfortunately EEG was not performed to not further delay the imaging or reperfusion treatment in the presence of disabling symptoms.

**Fig. 1:**
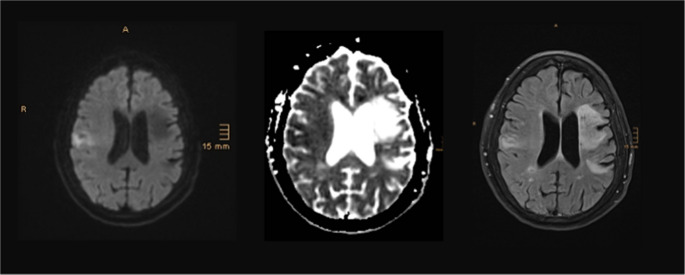
Axial MRI (left to right: DWI, ADC, FLAIR) demonstrating bilateral opercular infarction causing FCMS. DWI hyperintensity with corresponding hypointensity on ADC indicates acute stroke of the right opercular region with preexisting chronic infarct areas in the left hemisphere involving the left operculum

During the following 72 h, the trismus resolved spontaneously. Subsequent neurological examination revealed severe dysarthria, bilateral facial weakness, buccofacial apraxia and pronounced dysphagia. The facial paresis could be overcome when the patient laughed, revealing the cardinal symptom of Foix-Chavany-Marie syndrome (FCMS, see Video 1). MRI workup in the following days confirmed an acute infarction of the right operculum, gyrus praecentralis and insula of the right hemisphere and older infarctions in the territory of the left medial cerebral artery including the left frontal opercular region (see Fig. 1).

Continous in-hospital ECG monitoring as well as standardized long time ECG over 24 h showed no atrial fibrillation. In transthoracic and transesophageal echocardiography, no cardioembolic source was detected. No higher grade stenoses of the cerebral arteries could be detected apart from a previously described M1 stenosis on the left side. Laboratory testing showed elevated cardiolipin IgG antibodies but without fulfilling clinical criteria for antiphospholipid syndrome; therefore, the stroke was classified as embolic stroke of undetermined source.

With early intensive physiotherapy, speech and language as well as occupational therapy, our patient’s clinical symptoms improved. She was able to speak single words and simple sentences. Gradually, voluntary movements of the right corner of the mouth returned, and we saw a regredient left-sided hemiparesis. Still, the severe dysarthria and dysphagia persisted and, therefore, a percutaneous endoscopic gastrostomy was performed. After completion of acute diagnostic workup and treatment, we discharged our patient to a rehabilitation center. In the absence for a clear indication for oral anticoagulation or a dual antiplatelet regimen, secondary prophylactic treatment involved an antithrombotic monotherapy with ASS together with cardiovascular risk factor management including the enabled lipid-lowering therapy with rosuvastatin and ezetimibe.

## Discussion

This case sheds light on a rare presentation of an infrequently diagnosed neurovascular disease symptom. While acute onset hemiparesis, as in this case, is a common sign of ischemic stroke, trismus is rarely associated with ischemic cerebral injury and primarily caused by local muscle spasms due to regional or systemic infections (tetanus, rabies), side effects of regional anesthetics, jaw trauma or neoplasms.

Retrospectively, trismus was the first symptom of bilateral opercular syndrome (FCMS), though we discussed differential diagnoses of an epileptic seizure or a functional disorder. Together with the inability to properly inspect the oral cavity to rule out an active bleeding, this led to a delay of the thrombolytic therapy.

The supranuclear, voluntary control of the temporal, masseteric and pterygoid muscles is largely bilateral [2] and mediated through corticonuclear fibers originating in the anterior operculum descending through the genu of the internal capsule to the trigeminal nuclei in the brainstem. Bilateral damage of these regulating cortical areas could thus explain increased (spastic) tonicity of the effector muscles due to loss of cortical inhibition as in other forms of pseudobulbar palsy [3]. In our case, the clinical findings FCMS despite a unilateral acute right opercular stroke can most likely be explained by the pre-existing left hemispheric infarction involving the operculum, resulting in the pronounced pseudobulbar palsy with acute trismus.

Remarkably, in our patient trismus did not develop in the subacute or chronic stage but was instead the initial symptom of FCMS. In general, ischemic cerebral injuries initially present with decreased muscle tone in affected body parts and tend to lead to spasticity over weeks to months but there have been case reports of acute limb spasticity linked to stroke lesions in the anterior cingulate region and the superior frontal gyrus. To this date, few reports have linked acute onset trismus to bilateral damage of the operculum, the genu of the internal capsule or the pons [4, 5].

In our case, the trismus resolved spontaneously within 72 h while other case reports described the need for repeated injections of botulinumtoxin and administration of tizanidin to achieve symptom relief. The quick improvement of our patients’ symptoms shows that despite bilateral damage to a strategically important cerebral region, patients suffering from FCMS may profit from early and intensive multimodal therapy. Of course, the individual response to rehabilitation is difficult to predict since there is a high variability in long term outcomes of previously reported FCMS cases, ranging from early recovery in a matter of weeks up to persistent neurologic deficits.

Concerning the etiology of the patients subsequent stroke episodes, we had to classify an ESUS, although a cardioembolic source is highly probable concerning bihemispheric infarctions involving different vascular territories. Prolonged rhythm monitoring with an implantable loop recorder would be a reasonable next diagnostic step but could not be performed during the in-hospital diagnostic work-up.

## Conclusions

Acute onset trismus with anarthria can be due to stroke with Foix-Chavany-Marie syndrome especially with previous history of cerebrovascular injury of the frontal operculum. When the trismus resolves, characteristic clinical signs of autonomic-voluntary dissociation of the control of the oropharyngeal muscles together with typical imaging findings can confirm the diagnosis of FCMS. Knowing and understanding such unusual presentation can avoid a delay in acute stroke treatment.

## Supplementary Information

Below is the link to the electronic supplementary material.

Supplementary Material Video 1 – This Video shows the clinical examination of our patient several days after admission to our hospital. By then, the initial trismus had resolved, most likely spontaneously. Thus, a dissociation between autonomic and voluntary control of the lower facial muscles – a key finding of Foix-Chavany-Marie syndrome – become apparent. Not the absence of the facial paresis on the left side in the context of non-volitional motor activity, i.e. laughing.

Supplementary Material 2
