# Supplementary material for: Foix-Chavany-Marie syndrome with an unusual presentation: case report of a stroke with acute trismus
Source: Neurol Sci. 2026 May 14;47(6):493. doi: 10.1007/s10072-026-09107-z (PMC13176183; doi:10.1007/s10072-026-09107-z)
Supplement: Supplementary file 2 — Supplementary Material 2 [file 10072_2026_9107_MOESM2_ESM.pdf]

The CARE reporting checklist

For checking that clinical case report articles can be understood and used by everyone

	Item Description	Location (or reason for not reporting)
**Sections**		
1. Title	The area of focus and "case report" should appear in the title.	Heading, Page 1
2. Keywords	The key elements of this case in 2–5 words.	Paragraph 2 (after Abstract)
3. Abstract	3a – Introduction: What does this case add?<br>3b – Case presentation:<br>• The main symptoms of the patient(s).<br>• The main clinical findings.<br>• The main diagnoses and interventions.<br>• The main outcomes.<br>3c – Conclusion: What are the main "take-away" lessons from this case?	Paragraph 1
4. Introduction	Brief background summary of the case referencing the relevant medical literature.	Paragraph 3
5a. Patient information	5a – Demographic information of the patient (age, gender, ethnicity, occupation).<br>5b – Main symptoms of the patient (chief complaint).<br>5c – Medical, family, and psychosocial history—including lifestyle and genetic information whenever possible, details about relevant comorbidities, and past interv...	Paragraph 4 ("Case report")
6. Clinical findings	Describe the relevant physical examination (PE) findings.	Paragraph 4 ("Case report"), Video 1
7. Timeline	Depict important date and times in this case (table or figure).	Paragraph 4 ("Case report")
8. Diagnostic assessment	8a – Diagnostic methods (e.g., physical examination, laboratory testing, imaging, questionnaires)<br>8b – Diagnostic challenges (e.g., financial, language, or cultural)	Paragraph 4 ("Case report"), Figure 1

	8c – Diagnostic reasoning including other diagnoses considered<br><br>8d – Prognostic characteristics (e.g., staging) where applicable.	
9. Therapeutic Intervention	9a – Types of intervention (e.g., pharmacologic, surgical, preventive, self-care)<br><br>9b – Administration (e.g., dosage, strength, duration)<br><br>9c – Changes in intervention (with rationale).	Paragraph 4 (“Case report”)
10. Follow up and outcomes	10a – Clinician and patient-assessed outcomes<br><br>10b – Important follow-up test results (positive and negative)<br><br>10c – Intervention adherence and tolerability (and how this was assessed)<br><br>10d – Adverse and unanticipated events.	Paragraph 4 (“Case report”)
11. Discussion	Discussion (including conclusion):<br><br>11a – Strengths and limitations of the management of this case<br><br>11b – Relevant medical literature<br><br>11c – Rationale for conclusions (including assessment of cause and effect)<br><br>11d – Main “take-away” lessons of this case report.	Paragraph 5
12. Patient perspective	When appropriate patients should share their perspectives on the treatments they received.	Video 1
13. Informed consent	Did the patient give informed consent? Please provide if requested.	Provided Consent Form
